# Flood Inundation Mapping with Limited Observations Based on Physics-Aware Topography Constraint

**DOI:** 10.3389/fdata.2021.707951

**Published:** 2021-07-26

**Authors:** Arpan Man Sainju, Wenchong He, Zhe Jiang, Da Yan, Haiquan Chen

**Affiliations:** ^1^ Department of Computer Science, Middle Tennessee State University, Murfreesboro, TN, United States; ^2^ Department of Computer and Information Science and Engineering, University of Florida, Gainesville, FL, United States; ^3^ Department of Computer Science, University of Alabama at Birmingham, Birmingham, AL, United States; ^4^ California State University, Sacramento, CA, United States

**Keywords:** limited observation, physical constraint, spatial classification, machine learning, flood mapping

## Abstract

Spatial classification with limited observations is important in geographical applications where only a subset of sensors are deployed at certain spots or partial responses are collected in field surveys. For example, in observation-based flood inundation mapping, there is a need to map the full flood extent on geographic terrains based on earth imagery that partially covers a region. Existing research mostly focuses on addressing incomplete or missing data through data cleaning and imputation or modeling missing values as hidden variables in the EM algorithm. These methods, however, assume that missing feature observations are rare and thus are ineffective in problems whereby the vast majority of feature observations are missing. To address this issue, we recently proposed a new approach that incorporates physics-aware structural constraint into the model representation. We design efficient learning and inference algorithms. This paper extends our recent approach by allowing feature values of samples in each class to follow a multi-modal distribution. Evaluations on real-world flood mapping applications show that our approach significantly outperforms baseline methods in classification accuracy, and the multi-modal extension is more robust than our early single-modal version. Computational experiments show that the proposed solution is computationally efficient on large datasets.

## 1 Introduction

Given a spatial raster framework with explanatory feature layers, a spatial contextual layer (e.g., an elevation map), and a set of training samples with class labels outside the framework, the spatial classification problem aims to learn a model that can predict the class layer Du et al. (2019); Jiang (2019, 2020); Shekhar et al. (2015); Wang P. et al. (2018); Zhang et al. (2019); Karpatne et al. (2016); Jiang (2019). We particularly focus on spatial classification with limited feature observations, i.e., only limited pixel locations in the raster framework have explanatory feature data available. For example, observation-based flood inundation extent mapping aims to classify all pixels in the raster framework into flood and dry classes, even in the case whereby only a part of the pixels have corresponding spectral features. In this example, the elevation values are available for all pixels in the framework, but only limited pixel locations have spectral data (e.g., a drone or aerial plane could not cover the entire region due to limited time during a flood disaster).

The problem is important in many applications such as flood extent mapping. Flood extent mapping is crucial for disaster management, national water forecasting, and energy and food security Jiang and Shekhar (2017); Jiang et al. (2019); Xie et al. (2018). For example, during hurricane floods, first responders needed to know where the floodwater is in order to plan rescue efforts. In national water forecasting, accurate flood extent maps can be used to calibrate and validate the NOAA National Water Model National Oceanic and Atmospheric Administration (2018b). In current practice, flood extent maps are mostly produced by forecasting models, whose accuracy is often unsatisfactory in a high spatial resolution Cline (2009); Merwade et al. (2008). Other ways to generate flood maps involve sending a field crew on the ground (e.g., recording high watermarks), but the process is both expensive and time-consuming. A promising alternative is to utilize Earth observation data from remote sensors. However, sensor observations often have limited spatial coverage due to only a subset of sensors being deployed at certain spots, making it a problem of spatial classification with limited feature observations. For example, during a flood disaster, a drone can only collect spectral images in limited areas due to time limit. Though we use flood mapping as a motivation example, the problem is general for other applications such as water quality monitoring Yang and Jin (2010) in river networks.

The problem poses several unique challenges that are not well addressed by traditional classification techniques. First, there are limited feature observations on samples in the raster framework due to only a subset of sensors being deployed in certain regions. In other words, only a subset of samples have complete explanatory feature values, making it hard to predict classes for all samples. Second, among the sample pixels with complete explanatory feature values, their feature values may contain rich noise and obstacles (e.g., clouds and shadows). Third, the explanatory features of image pixels can be subject to class confusion due to heterogeneity. For instance, pixels of tree canopies above flood water have the same spectral features as trees in dry areas, yet the classes of these pixels are different. Finally, the number of pixel locations can be very large for high-resolution data (e.g., hundreds of millions of pixels). This requires scalable algorithms.

Over the years, various techniques have been developed to address missing feature observations (or incomplete data) in classification García-Laencina et al. (2010). Existing methods can be categorized into data cleaning or imputation, extending classification models to allow for missing values, and modeling missing features as hidden variables in the EM algorithm. Data cleaning will remove samples that miss critical feature values. Data imputation focuses on filling in missing values either by statistical methods Little and Rubin (2019) (e.g., mean feature values from observed samples) or by prediction models (e.g., regression) based on observed samples Batista and Monard (2002); Bengio and Gingras (1996); Rubin (2004); Schafer (1997); Yoon and Lee (1999). There are also approaches that handle missing values by the multi-task strategy (i.e., partition different patterns of missing values into different tasks) as in García-Laencina et al. (2013); Zhou et al. (2011); Wang et al. (2018a). Another approach focuses on classification models and algorithms that allow for missing feature values in learning and prediction without data imputation. For example, a decision tree model allows for samples with missing features in learning and classification Quinlan (1989, 2014); Webb (1998). During learning, for a missing feature value in a sample, a probability weight is assigned to each potential feature value based on its frequency in observed samples. During classification, a decision tree can explore all possible tree traversal paths for samples with missing features and select the final class prediction with the highest probability. Similarly, there are some other models that have been extended to allow for missing feature values, such as neural network ensembles Jiang et al. (2005), and support vector machine Chechik et al. (2007); Pelckmans et al. (2005); Smola et al. (2005). The last category is to model missing feature values as hidden variables and use the EM (Expectation-Maximization) algorithm for effective learning and inference Ghahramani Z. and Jordan M. I. (1994, 1994a); McLachlan and Krishnan (2007); Williams et al. (2007). Specifically, the joint distribution of all samples’ features (both observed and missing features) can be represented by a mixture model with fixed but yet unknown parameters. In the EM algorithm, we can use initialized parameters and observed features to estimate the posterior distribution of hidden variables (missing features), and then further update the parameters for the next iteration. However, all these existing methods assume that incomplete feature observations are rare and thus cannot be effectively applied to our problem where the vast majority of samples have missing features (i.e., limited feature observations).

To fill this gap, we recently proposed a new approach that incorporates physics-aware structural constraints into model representation Sainju et al. (2020b). Our approach assumes that a spatial contextual feature (elevation map) is fully observed for every sample location, and establishes the topography constraints (i.e., water flow directions across elevation contours) from the elevation map He and Jiang (2020); Jiang (2020). The advantage of such physical constraints is that it provides a global dependency structure of class labels (i.e., flood or dry) across locations beyond a spatial neighborhood. We design efficient algorithms for model parameter learning and class inference and conduct experimental evaluations to validate the effectiveness and efficiency of the proposed approach against existing works. Motivated by the observations that sample features can be heterogeneous with multiple modalities, this paper extends our recent approach by allowing for multi-modal feature distribution in each class. We also propose the parameter learning algorithms for the extended model. In summary, the paper makes the following contributions:

• We extend the model by allowing feature values of samples in each class to follow a multi-modal distribution.
• We evaluated the proposed model on two real-world hydrological datasets. Results show that the new multi-modal solution is more robust than the previous single-modal version especially when feature distribution in training samples is multi-modal.
• Computation experiments show that the proposed solution is scalable to a large data volume.

## 2 Problem Statement

### 2.1 Preliminaries

Here we define several basic concepts that are used in the problem formulation.

A *spatial raster framework* is a tessellation of a 2D plane into a regular grid of *N* cells. The framework can contain *m* explanatory feature layers (e.g., spectral bands in satellite imagery), a potential field layer (e.g., elevation), and a class layer (e.g., *flood* or *dry*).

Each pixel in a raster framework is a *spatial data sample*, denoted by $s_{n}=\left(x_{n},\phi_{n},y_{n}\right)$, where n∈ℕ,1≤n≤N, $x_{n}\in {\mathbb{R}}^{m\times 1}$ is a vector of *m* non-spatial explanatory feature values with each element corresponding to a feature layer, $\phi_{n}\in \mathbb{R}$ is a pixel’s potential field value, and $y_{n}\in \left\{0,1\right\}$ is a binary class.

A raster framework with all samples is denoted by $ℱ=\left\{s_{n}|n\in \mathbb{N},1\leq n\leq N\right\}$, non-spatial explanatory feature matrix of all samples are denoted by $X={\left[x_{1},\ldots ,x_{N}\right]}^{T}$, the potential field vector is denoted by $\Phi ={\left[\phi_{1},\ldots ,\phi_{N}\right]}^{T}$, and the class vector is denoted by $Y={\left[y_{1},\ldots ,y_{N}\right]}^{T}$.

In a raster framework, it may happen that only a limited number of samples have non-spatial explanatory features being observed. We define O as the set of indices for these fully observed samples. Samples with fully observed (complete) explanatory features are denoted by $\left\{x_{n}|n\in O\right\}$. Their feature matrix is denoted by $X_{o}$.

### 2.2 Problem Definition

**FIGURE 1:**
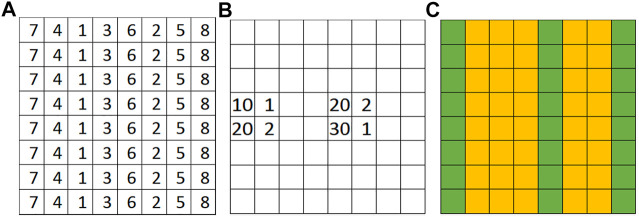
An illustration problem example. **(A)** Spatial potential field (elevation) **(B)** Partially observed non-spatial feature values **(C)** Ground truth classes (green for dry, orange for flood).

Given a raster framework with the explanatory features of a limited number of samples $X_{o}$, the potential field layer of all samples in the framework $\Phi ={\left[\phi_{1},\ldots ,\phi_{N}\right]}^{T}$, and a set of training pixels with class labels, the problem aims to learn a classifier *f* to predict the class layer $Y=f\left(X_{o},\Phi \right)$. For example, in flood extent mapping, the explanatory feature layers are spectral bands of earth imagery; the spatial contextual layer is geographic terrains based on elevation, and the target class layer are classes of flood or dry. We assume that the elevation values are available for all pixels in the framework (assuming that these values do not frequently change over time) but only limited pixel locations have spectral observations (e.g., a drone or aerial plane could not cover the entire region). Figure 1 shows a toy example of a raster framework that consists of 64 samples with a one-dimensional explanatory feature and a potential field layer. There are only eight samples with observed explanatory features (four non-empty cells in Figure 1B). The goal is to learn a model that can predict the class layer in Figure 1C.

## 3 Approach

In this section, we introduce our proposed approach. We start with physics-aware structural constraints and then introduce our probabilistic model and its learning and inference algorithms. We will introduce our approach in the context of the flood mapping application, but the proposed method can be potentially generalized to other applications such as material science Wales et al. (1998), Wales (2003) and biochemistry Edelsbrunner and Koehl (2005); Günther et al. (2014).

### 3.1 Physics-Aware Structural Constraint

**FIGURE 2:**
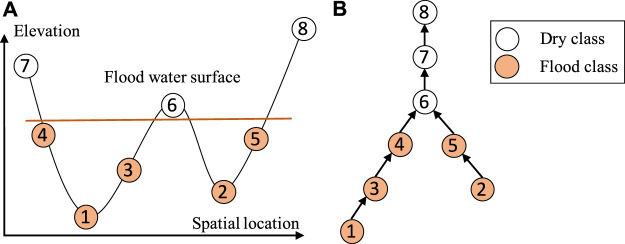
Illustration of partial order class dependency **(A)** Eight consecutive locations in 1D space **(B)** Partial order constraint in a reverse tree.

The main idea of our proposed approach is to establish a spatial dependency structure of sample class labels based on the physical constraint from the spatial potential field layers (e.g., water flow directions based on elevation). An illustration is provided in Figure 2. Figure 2A shows the elevation values of eight pixels in one dimensional space (e.g., pixels on a row in Figure 1). Due to gravity, water flows from high locations to nearby lower locations. If location 4 is flooded, locations 1 and 3 must also be flooded. Such a dependency structure can be established based on the topology of the potential field surface (e.g., elevation). Figure 2B shows a directed tree structure that captures the flow dependency structure. If any node is *flood*, then all sub-tree nodes must also be *flood* due to gravity. The structure is also called *split tree* in topology Carr et al. (2003); Edelsbrunner and Harer (2010), where a node represents a vertex on a mesh surface (spatial potential field) and an edge represents the topological relationships between vertices. We can efficiently construct the tree structure from a potential field map following the topological order of pixels based on the union-find operator (its time complexity is O(NlogN)) Carr et al. (2003). We omit details due to space limit. It is worth noting that although our illustrative example in Figure 2 is in one-dimensional space for simplicity, the structure is readily applicable to two-dimensional space Jiang and Sainju (2019). We can create a single tree structure for the entire elevation map in Figure 1A.

### 3.2 Model Probabilistic Formulation

**FIGURE 3:**
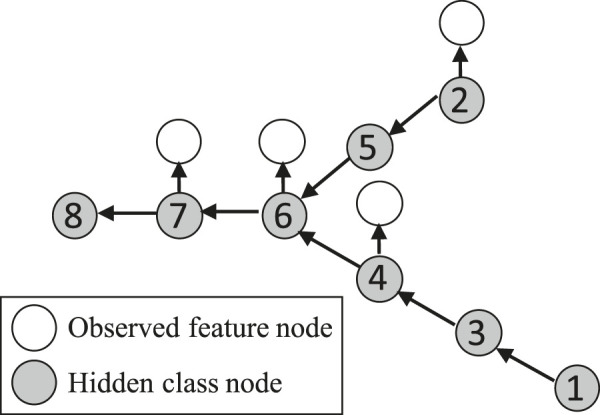
Illustration of model structure.

Now we introduce our approach that integrates physics-aware structural constraint into the probabilistic model formulation to handle limited feature observations. The overall idea of the model structure is similar to Xie et al. (2018); Jiang et al. (2019); Jiang and Sainju (2019); Sainju et al. (2020a); Jiang and Sainju (2021). Figure 3 illustrates the overall model structure. It consists of two layers: a hidden class layer with unknown sample classes ($y_{n}$) and an observation layer with limited sample feature vectors ($x_{n}$). Each node is a spatial data sample (raster pixel). Edge directions represent a dependency structure based on physical constraints.

The joint distribution of all samples’ features and classes are in Eq. 1, where $P_{n}$ is the set of parent nodes in the dependency tree, and $y_{k\in P_{n}}\equiv \left\{y_{k}|k\in P_{n}\right\}$ is the set of parent classes. For a leaf node *n*, $P_{n}=\emptyset$, and $P\left(y_{n}|y_{k\in P_{n}}\right)=P\left(y_{n}\right)$.

$$P\left(X_{o},Y\right)=P\left(X_{o}|Y\right)P\left(Y\right)=\prod_{n\in O}P\left(x_{n}|y_{n}\right)\prod_{n=1}^{N}P\left(y_{n}|y_{k\in P_{n}}\right)$$ (1)

The sample feature distribution in each class is assumed i.i.d. Gaussian for simplicity, as shown in Eq. 2, where $\mu_{y_{n}}$ and $\Sigma_{y_{n}}$ are the mean and covariance matrix of feature vector $x_{n}$ for class $y_{n}$ ($y_{n}=0,1$).

$$P\left(x_{n}|y_{n}\right)∼N\left(\mu_{y_{n}},\Sigma_{y_{n}}\right)$$ (2)

The class transitional probability follows a partial order. For instance, because of gravity, if any parent’s class is *dry*, the child’s class must be *dry*. On the other hand, if all parents’ classes are *flood*, then the current child’s class has a high probability of being *flood* too. Consider *flood* as class 1 and *dry* as class 0, then the previous assumption is actually conditioned on the product of parent classes $y_{P_{n}}\equiv \prod_{k\in P_{n}}y_{k}$, as expressed in Table 1, where π and ρ are parameters for class transitional probability and class prior probability.

**TABLE 1:** Class transition probability and prior probability.

$P\left(y_{n}|y_{P_{n}}\right)$	$y_{P_{n}}=0$	$y_{P_{n}}=1$
$y_{n}=0$	1	1−ρ
$y_{n}=1$	0	ρ
	$P\left(y_{n}\right)$	
$y_{n}=0$	1−π	
$y_{n}=1$	π	

### 3.3 Model Parameter Learning and Class Inference

Model parameters consist of the mean and covariance matrix of features in each class, the prior class probability, and the class transition probability. We denote the entire set of parameters as $\Theta =\left\{\rho ,\pi ,\mu_{c},\Sigma_{c}|c=0,1\right\}$. Learning parameters poses two challenges: first, Eq. 1 contains both unknown parameters and hidden class variables $Y={\left[y_{1},\ldots ,y_{N}\right]}^{T}$ that are non-i.i.d.; second, the number of samples (*N*) can be huge (e.g., millions of pixels).

We use an expectation-maximization (EM) algorithm together with message (belief) propagation. The main idea of the EM algorithm is to first initialize a parameter setting, and compute the posterior expectation of log-likelihood (Eq. 1) on hidden class variables (E-step). The posterior expectation is a function of unknown parameters. Thus, we can update parameters by maximizing the posterior expectation (M-step). The two steps can repeat iteratively until the parameter values converge. One remaining issue is the calculation of posterior expectation of log-likelihood on hidden class variables. This requires to compute the marginal posterior distribution of $P\left(y_{n},y_{k\in P_{n}}|O,\Theta_{0}\right)$ and $P\left(y_{n}\right)$ for each node $s_{n}$. This is very challenging due to the high dimensionality of Y. To address this challenge, we use message propagation. For message propagation, we use the sum and product algorithm Kschischang et al. (2001); Ronen et al. (1995). Propagation of message along tree nodes is similar to marginalizing out variables in the joint distribution in Eq. 1. Due to the space limit, we only show the major steps in the following discussion.

The message passing process is based on the sum-product algorithm, which involves tree traversal operations. After parameters learning, we can infer class variables by maximizing the joint probability. We use a dynamic programming algorithm called *max-sum* Rabiner (1989). It is similar to the above sum and product algorithm. The main difference is that we replace the *sum* operation with a *max* operation during message propagation.

### 3.4 Intuitions on How the Model Works

The main intuition behind how our model handles limited observations is that the model can capture physical constraints between sample classes. The spatial structural constraints are derived from the potential field layer that is fully observed on the entire raster framework, regardless of whether non-spatial features are available or not. The topological structure in a split tree is consistent with the physical law of water flow directions on a topographic surface based on gravity. In this sense, even though many samples in the raster framework do not have non-spatial explanatory features observed, we can still infer their classes based on information from the pixels in the upstream or downstream locations.

Another potential question is how our model can effectively learn parameters given very limited observations. This question can be answered from the perspective of how model learning works. The major task of model learning is to effectively update parameters of $P\left(x_{n}|y_{n}\right)$ for observed pixels in the test region, so that we can infer the posterior class probabilities on these pixels and further infer hidden classes on other pixels. As long as the training samples could give the model a reasonable initial estimate of posterior class probabilities on the observed pixels (e.g., truly dry pixels having a higher probability of being dry), the update of parameters should be effective. This is because that parameter updates are largely weighted average of the sample mean and covariance matrices on fully observed pixels. The corresponding weights are the posterior class probability of observed samples.

### 3.5 Extension to Multi-modal Feature Distribution

This subsection introduces an extension to our proposed model. In the conference version, the joint distribution of all sample features and classes follow a conditional independence assumption based on the tree structure derived from physical constraint (Eq. 1). Sample feature distribution in each class is assumed i.i.d. Gaussian (Eq. 2). In real-world datasets, the actual sample feature distribution in each class can be multi-modal, violating the earlier assumption on a single-modal Gaussian distribution. To account for this observation, we extend our model to allow for multi-modal sample feature distribution in each class. Specifically, we assume that sample features follow an i.i.d. mixture of Gaussian distribution.

For the extended model, the joint distribution of all observed samples’ features and classes can be expressed the same way as Eq. 1. The joint probability can be decomposed into local factors, i.e., the conditional distribution of features in each class and class transitional probability. The assumption on class transitional probability for non-leaf nodes and prior class probability for leaf nodes remain the same as before (Table 1). What is different is the conditional probability of sample feature vector given its class. In the extended multi-modal model, sample feature in each class is assumed i.i.d. mixture Gaussian distribution, as shown in Eq. 3, where $\mu_{y_{n}}^{i}$ and $\Sigma_{y_{n}}^{i}$ are the mean and covariance matrix of the *i*-th Gaussian component of feature vector $x_{n}$ for class $y_{n}$ ($y_{n}=0,1$), $\phi_{y_{n}}^{i}$ is the probability of a sample in class $y_{n}$ belonging to the *i*th Gaussian component $N\left(\mu_{y_{n}}^{i},\Sigma_{y_{n}}^{i}\right)$, $K_{y_{n}}$ is the number of Gaussian components (modes) in the feature distribution of samples in class $y_{n}$.

$$P\left(x_{n}|y_{n}\right)∼\sum_{i=1}^{K_{y_{n}}}\phi_{y_{n}}^{i}N\left(\mu_{y_{n}}^{i},\Sigma_{y_{n}}^{i}\right),y_{n}=0,1$$ (3)

Based on the extended probabilistic formulation, we can use the same EM algorithm with message propagation for parameter learning. The entire set of parameters can be denoted as $\Theta =\left\{\rho ,\pi ,\phi_{c}^{i},\mu_{c}^{i},\Sigma_{c}^{i}|1\leq i\leq K_{c},c=0,1\right\}$. The EM algorithm involves iterations with each iteration consisting of two major steps: an E-step that calculates the marginal class posterior probabilities by message propagation based on old parameters, and an M-step that finds the optimal parameters to maximize the overall objective. We can use message propagation based on the sum and product algorithm Kschischang et al. (2001); Ronen et al. (1995). The main difference is that the calculation of messages related to the factor $P\left(x_{n}|y_{n}\right)$ is based on the mixture of Gaussian distribution instead of a single-modal Gaussian distribution, as specified in Eq. 3.

After calculating the marginal posterior, we update model parameters by maximizing the posterior expectation of log-likelihood. Since we extend the probabilistic formulation of feature distribution to a mixture of Gaussian, we also need to revise the parameter update formula. The extended parameter update formulas are shown by equations below. The symbol $\Theta_{0}$ represents the old parameter values in the previous iteration of the EM algorithm, which is used in the calculation of messages and estimated posterior class probabilities. These calculated terms are then used to update the parameters in the formulas below to get a new Θ (the maximization step or M-step in the EM algorithm).

$$\rho =\frac{\sum_{n|P_{n}\neq \emptyset }\sum_{y_{n}}\sum_{y_{P_{n}}}y_{P_{n}}\left(1-y_{n}\right)P\left(y_{n},y_{P_{n}}|X,\Theta_{0}\right)}{\sum_{n|P_{n}\neq \emptyset }\sum_{y_{n}}\sum_{y_{P_{n}}}y_{P_{n}}P\left(y_{n},y_{P_{n}}|X,\Theta_{0}\right)}$$ (4)

$$\pi =\frac{\sum_{n|P_{n}=\emptyset }\sum_{y_{n}}y_{n}P\left(y_{n}|X,\Theta_{0}\right)}{\sum_{n|P_{n}=\emptyset }\sum_{y_{n}}P\left(y_{n}|X,\Theta_{0}\right)}$$ (5)

$$\phi_{c}^{i}=\frac{\sum_{n\in O}P\left(y_{n}=c|X,\Theta_{0}\right)\gamma_{c}^{i}\left(x_{n},\Theta_{0}\right)}{\sum_{i=1}^{K_{c}}\sum_{n\in O}P\left(y_{n}=c|X,\Theta_{0}\right)\gamma_{c}^{i}\left(x_{n},\Theta_{0}\right)},c=0,1$$ (6)

$$\mu_{c}^{i}=\frac{\sum_{n\in O}x_{n}P\left(y_{n}=c|X,\Theta_{0}\right)\gamma_{c}^{i}\left(x_{n},\Theta_{0}\right)}{\sum_{n\in O}P\left(y_{n}=c|X,\Theta_{0}\right)\gamma_{c}^{i}\left(x_{n},\Theta_{0}\right)},c=0,1$$ (7)

$${\text{Σ}}_{c}^{i}=\frac{\sum_{n\in O}\left(x_{n}-\mu_{c}\right){\left(x_{n}-\mu_{c}\right)}^{T}P\left(y_{n}=c|X,\Theta_{0}\right)\gamma_{c}^{i}\left(x_{n},\Theta_{0}\right)}{\sum_{n\in O}P\left(y_{n}=c|X,\Theta_{0}\right)\gamma_{c}^{i}\left(x_{n},\Theta_{0}\right)},c=0,1,$$ (8)

$$\text{where}\;\gamma_{c}^{i}\left(x_{n},\Theta_{0}\right)=\frac{\phi_{0,c}^{i}N\left(x_{n}|\mu_{0,c}^{i},\Sigma_{0,c}^{i}\right)}{\sum_{i=1}^{K_{c}}\phi_{0,c}^{i}N\left(x_{n}|\mu_{0,c}^{i},\Sigma_{0,c}^{i}\right)},c=0,1,$$ (9)

In the new parameter update formulas, the formulas for ρ and π are similar to the single-modal version. The main difference is related to parameters for feature distributions, such as $\phi_{c}^{i}$, $\mu_{c}^{i}$ and ${\text{Σ}}_{c}^{i}$ in Eqs 6–8. The key difference is the additional weighting terms $\gamma_{c}^{i}\left(x_{n},\Theta_{0}\right)$ in Eq. 9. Intuitively, the intermediate variable $\gamma_{c}^{i}\left(x_{n},\Theta_{0}\right)$ reflects the weight of a sample with feature $x_{n}$ belong to the *i*-th Gaussian component of class *c*. For example, in the parameter update formula for $\mu_{c}^{i}$ (Eq. 7), the updated mean for features in the *i*-th component of class *c* is based on the feature vector of each sample, averaged by the sample’s weight on the *i*-th component of class *c* ($\gamma_{c}^{i}\left(x_{n},\Theta_{0}\right)$). Similar interpretation can be given on the update formulas for $\phi_{c}^{i}$ and ${\text{Σ}}_{c}^{i}$.

Another issue is the parameter initialization before the EM iteration, the initial parameters of $\phi_{c}^{i}$, $\mu_{c}^{i}$ and ${\text{Σ}}_{c}^{i}$ can be initialized based on a small set of training samples (pixels) with known labels. This can be done by an EM clustering for feature values in each class. The cluster centroids can be initialized by randomly select $K_{c}$ samples from the training samples in class *c*.

After parameter learning, we can use the same class inference algorithm based on message propagation (*max-sum* Rabiner (1989)). The calculation of messages is similar to before, except that the message related to $P\left(x_{n}|y_{n}\right)$ are calculated based on the mixture of Gaussian distribution.

## 4 Experimental Evaluation

### 4.1 Experiment Setup

In this section, we compared our proposed approach with baseline methods in related works on real-world datasets. Evaluation candidate methods are listed below. Note that we did not include data imputation methods (e.g., filling in mean feature values) due to its low capability of handling very limited observations. We used default parameters for open source tools. Experiments were conducted on a Dell workstation with Intel(R) Xeon(R) CPU E5-2687w v4 @ 3.00GHz, 64 GB main memory, and Windows 10.

• **Label propagation with structure (LP-Structure)**: In the implementation of this baseline method, we used the maximum likelihood classifier (MLC) and gradient boosted model (GBM) respectively to pre-classify fully observed samples and then ran label propagation Wang and Zhang (2007) on the topography tree structure. We named them as **LP-Structure-MLC** and **LP-Structure-GBM.** The initial classifiers were from R packages.
• **EM with i.i.d. assumption (EM-i.i.d.)**: In the implementation of this baseline method Ghahramani Z. and Jordan M. (1994), we treated missing features and unknown classes as latent variables and used the EM algorithm assuming that sample features follow i.i.d. Gaussian distribution in each class. Moreover, we assumed RGB (red, green, blue) features and elevation features are uncorrelated.
• **EM with structure**: This is our proposed approach. We treated unknown classes as latent variables and used the EM algorithm assuming that samples follow the topography tree dependency structure. The codes were implemented in C++. There are two configurations: single-modal feature distribution (**EM-Structure-Single**) and multi-modal feature distribution (**EM-Structure-Multi**).

*Data Description:* Our real-world data were collected from Kinston and Grimesland in North Carolina, 2016. The data include aerial imagery from NOAA National Geodetic Survey National Oceanic and Atmospheric Administration (2018a) with red, green, blue bands in a 2-m resolution and a digital elevation map from the University of North Carolina Libraries NCSU Libraries (2018). The test region size was 1743 by 1,349 pixels in Kinston and 2,757 by 3,853 pixels in Grimesland. The number of observed pixels was 31,168 in Kinston and 237,312 in Grimesland. The numbers of training and testing pixels are provided in Table 2.

**TABLE 2:** Dataset description.

Dataset	Training Set		Testing Set	
	**Dry**	**Flood**	**Dry**	**Flood**
Matthew, Kinston	5,000	5,000	48,071	47,967
Matthew, Grimesland	5,000	5,000	75,670	59,405

*Evaluation Metrics*: For classification performance evaluation, we used precision, recall, and F-score. For computational performance evaluation, we measured the running time costs in seconds.

### 4.2 Classification Performance Evaluation

We first compared methods on precision, recall, and F-score on the two real-world datasets. The results were summarized in Tables 3, 4 respectively. On the Kinston dataset, EM algorithm with the i.i.d. assumption performed the worst with an average F-score of 0.66. The reason was that this method was not able to utilize the spatial structural constraint between sample classes. Its training process only updated the parameter of Gaussian feature distribution in each class. When predicting the classes of samples with only elevation feature, the method used only the learned Gaussian distribution of elevation feature on each class without considering spatial structure based on elevation values. On the same dataset, label propagation after pre-classification with the GBM model and the maximum likelihood classifier slightly outperformed the EM algorithm with the i.i.d. assumption. The main reason was that label propagation on the topography tree (split tree) structure utilized the physical constraint between sample classes when inferring the classes of unobserved samples without RGB features. However, label propagation still showed significant errors, particularly in the low recall on the dry class. Through analyzing the predicted map, we observed that the label propagation algorithm was very sensitive to the pre-classified class labels on the observed samples in the test region. Errors in the pre-classification phase may propagation into unobserved samples (those without RGB feature values). In label propagation methods, once the errors were propagated to unobserved samples, they were hard to be reverted. This was different from the EM algorithm, which could update the probabilities in iterations. We did not report the results of label propagation on a grid graph structure (only considering spatial neighborhood structure without physics-aware constraint) due to poor results. Our model based on the EM algorithm assuming structural dependency between class labels performed the best with an average F-score of 0.96. The main reason was that our model could leverage the physical constraint to infer unobserved samples, and also could effectively update sample probabilities during iterations with the EM algorithm. In our model, we used training samples to initialize the parameters of the Gaussian distribution of sample features in each class. Based on the reasonable initial parameters, we can have a reasonable estimation of the posterior class probabilities of all samples in the test region. Based on the posterior class probabilities, the distribution parameters could be further updated. The representative training samples helped make sure that parameter iterations would converge in the right path.

**TABLE 3:** Comparison on Mathew, Kinston flood data.

Classifiers	Class	Prec.	Recall	F	Avg. F
LP-Structure-GBM	Dry	0.91	0.56	0.69	0.74
	Flood	0.68	0.94	0.79	
LP-Structure-MLC	Dry	0.86	0.55	0.67	0.72
	Flood	0.67	0.91	0.77	
EM-i.i.d.	Dry	1.00	0.39	0.56	0.66
	Flood	0.62	1.00	0.76	
EM-Structure-Single	Dry	0.94	0.99	0.96	0.96
	Flood	0.99	0.94	0.96	

**TABLE 4:** Comparison on Mathew, Grimesland flood data.

Classifiers	Class	Prec.	Recall	F	Avg. F
LP-Structure-GBM	Dry	0.81	0.60	0.69	0.70
	Flood	0.61	0.82	0.70	
LP-Structure-MLC	Dry	0.90	0.75	0.82	0.81
	Flood	0.73	0.90	0.81	
EM-i.i.d.	Dry	0.83	0.74	0.78	0.77
	Flood	0.71	0.80	0.75	
EM-Structure-Single	Dry	0.99	0.96	0.97	0.97
	Flood	0.95	0.99	0.97	

Similar results were observed on the Grimesland dataset. In the label propagation method, pre-classification based on GBM performed worse than pre-classification based on MLC. The reason may be due to overfitting of GBM compared with MLC when predicting initial labels on the fully observed samples. The EM algorithm with the i.i.d. assumption performed slightly better on this dataset. The reason is likely that the final prediction of classes of the unobserved samples (with only elevation feature but without RGB features) was based on a slightly better fitted normal distribution. Our model showed the best performance with an F-score of 0.97.

#### 4.2.1 The Effect of Model Initial Parameters

**FIGURE 4:**
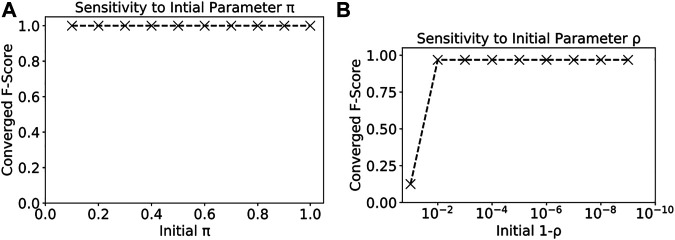
Sensitivity of our model to initial parameters π and ρ.

We also analyzed the sensitivity of our proposed model on different initial parameter settings. The parameters of $\mu_{c}$ and $\Sigma_{c}$ were estimated from training data, but parameters ρ and π were from user input. Since ρ captured the transitional probability of a sample being flood given its parents were all flood, its value should be very high (close to 1) due to spatial autocorrelation. π is the initial class prior probabilities for samples without parent nodes (local lowest location). We could set it close to 0.5. We tested the sensitivity of our model to different initial values of ρ and π on the Kinston dataset. We first fixed ρ as 0.999 and varied the value of π from 0.1 to 0.9. Then we fixed π as 0.3 and varied the value of ρ from 0.9. The results were shown in Figure 4. We can see that the model was generally not very sensitive to the initial parameter values. For parameter ρ, as long as 1−ρ was smaller than 0.01 (ρ greater than 0.99), the converged F-score was good. For parameter π, the results were consistently good for our model with an initial π between 0.1 and 0.9. The main reason was that π influenced only a small number of samples at the local lowest locations on the elevation map.

**FIGURE 5:**
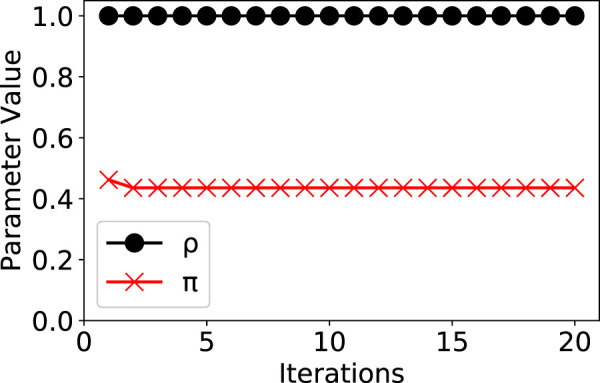
Parameter iterations and convergence in our model.

The parameter iterations of our model were shown in Figure 5. The model converged fast with only 20 iterations. Due to the space limit, we only show the iterations of parameters ρ and π.

### 4.3 Computational Performance Evaluation

**FIGURE 6:**
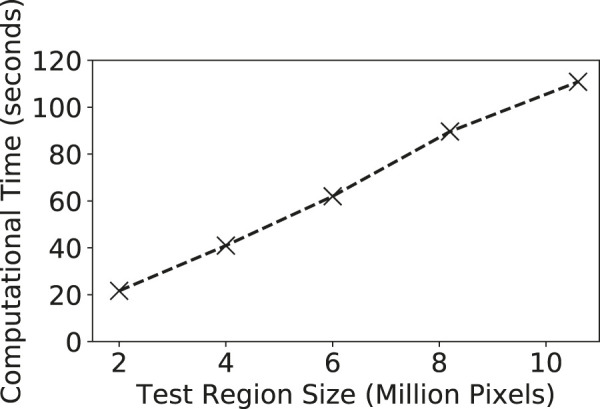
Computational performance of out model on varying test region sizes.

We also evaluated the computational performance of our model learning and inference on different input data sizes. We used the Grimesland dataset to test the effect of different test region sizes. We varied the region size from around 2 million pixels to over 10 million pixels. The computational time costs of our model were shown in Figure 6. It can be seen that the time cost grows almost linearly with the size of the test region. This was because our learning and inference algorithms involve tree traversal operations with a linear time complexity on the tree size (the number of pixels on the test region). The model is computationally efficient. It classified around 10 million pixels in around 2 min.

We further analyzed the time costs of different components in our model, including split tree construction, model parameter learning, and class inference. The results are summarized in Table 5. We analyzed the results on both datasets (same as the settings in Tables 3, 4. Results showed that tree construction and class inference took less time than parameter learning. This was because the learning involves multiple iterations of message propagation (tree traversal operations).

**TABLE 5:** Time costs of different components of our model on two datasets (seconds)

	Kinston	Grimesland
Tree construction part	3.2	8.39
Parameter learning part	25.74	86.79
Class inference part	3.8	15.62
Total time costs	32.74	110.80

### 4.4 Additional Comparison of EM Structured Between Single-modal and Multi-modal

This subsection provides additional evaluations on the comparison of our EM structured algorithms between the single-modal feature distribution (conference version) and multi-modal feature distribution (journal extension). In previous experiments, we found that our EM structured (single-modal) outperformed several baseline methods when feature distribution among training samples in each class is single-modal. In this experiment, we used the same two test regions and observed polygons as previous experiments (Table 2), but collected training samples that exhibit multi-modal feature distributions in each class. The new training dataset still contains 5,000 samples for dry class and 5,000 samples for flood class.

For both single-modal and multi-modal, we fixed the parameter π (prior class probability for leaf nodes) as 0.5, ρ as 0.999 (class transitional probability between a node and its parents) and the maximum number of iterations in EM as 40. We compared different candidate methods on their precision, recall, and F-score on the two test regions.

The new results were summarized in Tables 6, 7. On Kinston flood data, we can see that the performance of the three baseline methods was poor (F-score below 0.72). Among these methods, GBM with label propagation (LP) was worse (F-score around 0.34), likely due to serious overfitting issues from the varying feature distributions between the training and test samples. The performance of the proposed EM-structure model with single-modal feature distribution (conference version) significantly degraded (F-score dropped to 0.34). The reason was probably that the feature values of training samples follow a multi-modal distribution, violating the original assumption that features are single-modal Gaussian in each class. Thus, the parameter iterations were not ineffective during the learning iterations on observed samples in the test region. In contrast, the EM-structured with multi-modal distribution was more robust with the best performance (F-score around 0.97). Similar results were seen in the Grimesland dataset. The three baseline methods generally performed poorly, except for the label propagation with the maximum likelihood method achieving an F-score of 0.91. The reason was likely that the maximum likelihood classifier was simple and less prone to overfitting in initial label prediction. The EM-structured model with a single modal performed poorly (F-score around 0.28) due to the wrong assumption on feature distribution. In contrast, the EM-structured model with a multi-modal assumption performed the best with an F-score around 0.95.

**TABLE 6:** Comparison on Mathew, Kinston flood data.

Classifiers	Class	Prec.	Recall	F	Avg. F
LP-Structure-GBM	Dry	0.44	0.73	0.55	0.34
	Flood	0.26	0.09	0.14	
LP-Structure-MLC	Dry	0.85	0.56	0.67	0.72
	Flood	0.67	0.90	0.77	
EM-i.i.d.	Dry	1.00	0.40	0.57	0.67
	Flood	0.62	1.00	0.77	
EM-Structure-Single	Dry	0.50	1.00	0.66	0.34
	Flood	1.00	0.01	0.01	
EM-Structure-Multi (Our extension)	Dry	0.94	0.99	0.97	0.97
	Flood	0.99	0.94	0.97	

**TABLE 7:** Comparison on Mathew, Grimesland flood data.

Classifiers	Class	Prec.	Recall	F	Avg. F
LP-Structure-GBM	Dry	0.74	0.53	0.62	0.71
	Flood	0.75	0.88	0.81	
LP-Structure-MLC	Dry	0.84	0.96	0.90	0.91
	Flood	0.97	0.89	0.93	
EM-i.i.d.	Dry	0.57	0.87	0.69	0.70
	Flood	0.88	0.590	0.70	
EM-Structure-Single	Dry	0.38	1.00	0.55	0.28
	Flood	1.00	0.00	0.00	
EM-Structure-Multi (Our extension)	Dry	0.91	0.96	0.94	0.95
	Flood	0.97	0.94	0.96	

**FIGURE 7:**
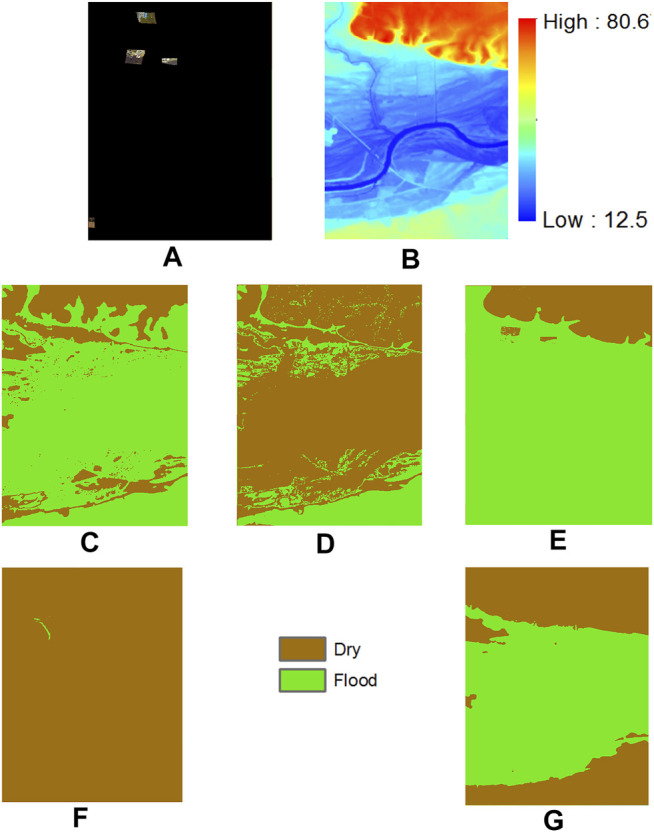
Results on Matthew flood, Kinston, NC **(A)** Limited observation aerial imagery in Kinston NC **(B)** Digital elevation model **(C)** LP-Structure-GBM result **(D)** LP-Strucrure-MLC result **(E)** EM-i.i.d. result **(F)** EM-Structure-Single result **(G)** EM-Structure-Multi result.

We also visualize the predicted class maps on the Kinston dataset in Figure 7 for interpretation. The input limited feature observations on spectral pixels in the test region are shown in Figure 7A. The observed pixels cover parts of the boundary of the flood region in the test region. The digital elevation image that was used to construct a tree structure based on physical constraint is shown in Figure 7B. From the topography of the area, we can *see* that the test region has a floodplain alongside a river channel that is spreading through the lower half of the image. The top area of the image have higher elevation. The predictions of label propagation on top of GBM (LP-Structure-GBM) and MLC (LP-Structure-MLC) are in Figures 7C,D. We can *see* a significant amount of misclassification (e.g., the flood area in the middle of Figure 7C is mistakenly predicted as dry, the dry area at the bottom of Figure 7B is mistakenly classified as flood). The reason is probably that initial class predictions by these models are noisy and these noisy labels further spread out during label propagation. The EM i.i.d. algorithm also shows significant errors in the bottom part of the image, where the elevation is lower. The reason is probably that the learned decision boundary on the elevation feature in the EM i.i.d. algorithm is inaccurate due to limited sample observations. The EM structured model with single-modal feature distribution performs poorly, classifying almost the entire area as dry. The reason is likely that the feature distribution in the model during parameter learning iterations is wrong, making the inferred classes largely wrong. In contrast, the EM structured model with multi-modal feature distribution identified the complete flood boundary.

## 5 Conclusions and Future Work

In this paper, we investigate the flood extent mapping application of spatial classification in a case whereby samples have limited feature observations. We extend our recent approach that incorporates physics-aware structural constraints (e.g., water flow directions on geographic terrains) into model structural representation. We propose efficient algorithms for model parameter learning and class inference. The extended model allows for multi-modal feature distribution with the mixture Gaussian model. Evaluations on flood mapping datasets show that the proposed approach outperformed existing methods in classification accuracy.

In future work, we plan to extend our proposed model to address other problems such as integrating noisy and incomplete observations such as volunteered geographic information (VGI). We also plan to explore incorporating deep learning into our framework to learn more complex feature distributions.

## Data Availability

Publicly available datasets were analyzed in this study. This data can be found here: https://geodesy.noaa.gov/storm_archive/storms/matthew/index.html.
